# Combined Impact of Health Behaviours and Mortality in Men and Women: The EPIC-Norfolk Prospective Population Study

**DOI:** 10.1371/journal.pmed.0050012

**Published:** 2008-01-08

**Authors:** Kay-Tee Khaw, Nicholas Wareham, Sheila Bingham, Ailsa Welch, Robert Luben, Nicholas Day

**Affiliations:** 1 Department of Public Health and Primary Care, Institute of Public Health, University of Cambridge School of Clinical Medicine, Cambridge, United Kingdom; 2 Medical Research Council, Epidemiology Unit, Cambridge, United Kingdom; 3 Medical Research Council, Dunn Nutrition Unit, Cambridge, United Kingdom; The University of Queensland, Australia

## Abstract

**Background:**

There is overwhelming evidence that behavioural factors influence health, but their combined impact on the general population is less well documented. We aimed to quantify the potential combined impact of four health behaviours on mortality in men and women living in the general community.

**Methods and Findings:**

We examined the prospective relationship between lifestyle and mortality in a prospective population study of 20,244 men and women aged 45–79 y with no known cardiovascular disease or cancer at baseline survey in 1993–1997, living in the general community in the United Kingdom, and followed up to 2006. Participants scored one point for each health behaviour: current non-smoking, not physically inactive, moderate alcohol intake (1–14 units a week) and plasma vitamin C >50 mmol/l indicating fruit and vegetable intake of at least five servings a day, for a total score ranging from zero to four. After an average 11 y follow-up, the age-, sex-, body mass–, and social class–adjusted relative risks (95% confidence intervals) for all-cause mortality(1,987 deaths) for men and women who had three, two, one, and zero compared to four health behaviours were respectively, 1.39 (1.21–1.60), 1.95 (1.70–-2.25), 2.52 (2.13–3.00), and 4.04 (2.95–5.54) *p* < 0.001 trend. The relationships were consistent in subgroups stratified by sex, age, body mass index, and social class, and after excluding deaths within 2 y. The trends were strongest for cardiovascular causes. The mortality risk for those with four compared to zero health behaviours was equivalent to being 14 y younger in chronological age.

**Conclusions:**

Four health behaviours combined predict a 4-fold difference in total mortality in men and women, with an estimated impact equivalent to 14 y in chronological age.

## Abstract 2

From a large prospective population study, Kay-Tee Khaw and colleagues estimate the combined impact of four behaviors--not smoking, not being physically inactive, moderate alcohol intake, and at least five vegetable servings a day--amounts to 14 additional years of life.

## Editors' Summary

**Background.:**

Every day, or so it seems, new research shows that some aspect of lifestyle—physical activity, diet, alcohol consumption, and so on—affects health and longevity. For the person in the street, all this information is confusing. What is a healthy diet, for example? Although there are some common themes such as the benefit of eating plenty of fruit and vegetables, the details often differ between studies. And exactly how much physical activity is needed to improve health? Is a gentle daily walk sufficient or simply a stepping stone to doing enough exercise to make a real difference? The situation with alcohol consumption is equally confusing. Small amounts of alcohol apparently improve health but large amounts are harmful. As a result, it can be hard for public-health officials to find effective ways to encourage the behavioral changes that the scientific evidence suggests might influence the health of populations.

**Why Was This Study Done?:**

There is another factor that is hindering official attempts to provide healthy lifestyle advice to the public. Although there is overwhelming evidence that individual behavioral factors influence health, there is very little information about their combined impact. If the combination of several small differences in lifestyle could be shown to have a marked effect on the health of populations, it might be easier to persuade people to make behavioral changes to improve their health, particularly if those changes were simple and relatively easy to achieve. In this study, which forms part of the European Prospective Investigation into Cancer and Nutrition (EPIC), the researchers have examined the relationship between lifestyle and the risk of dying using a health behavior score based on four simply defined behaviors—smoking, physical activity, alcohol drinking, and fruit and vegetable intake.

**What Did the Researchers Do and Find?:**

Between 1993 and 1997, about 20,000 men and women aged 45–79 living in Norfolk UK, none of whom had cancer or cardiovascular disease (heart or circulation problems), completed a health and lifestyle questionnaire, had a health examination, and had their blood vitamin C level measured as part of the EPIC-Norfolk study. A health behavior score of between 0 and 4 was calculated for each participant by giving one point for each of the following healthy behaviors: current non-smoking, not physically inactive (physical inactivity was defined as having a sedentary job and doing no recreational exercise), moderate alcohol intake (1–14 units a week; a unit of alcohol is half a pint of beer, a glass of wine, or a shot of spirit), and a blood vitamin C level consistent with a fruit and vegetable intake of at least five servings a day. Deaths among the participants were then recorded until 2006. After allowing for other factors that might have affected their likelihood of dying (for example, age), people with a health behavior score of 0 were four times as likely to have died (in particular, from cardiovascular disease) than those with a score of 4. People with a score of 2 were twice as likely to have died.

**What Do These Findings Mean?:**

These findings indicate that the combination of four simply defined health behaviors predicts a 4-fold difference in the risk of dying over an average period of 11 years for middle-aged and older people. They also show that the risk of death (particularly from cardiovascular disease) decreases as the number of positive health behaviors increase. Finally, they can be used to calculate that a person with a health score of 0 has the same risk of dying as a person with a health score of 4 who is 14 years older. These findings need to be confirmed in other populations and extended to an analysis of how these combined health behaviors affect the quality of life as well as the risk of death. Nevertheless, they strongly suggest that modest and achievable lifestyle changes could have a marked effect on the health of populations. Armed with this information, public-health officials should now be in a better position to encourage behavior changes likely to improve the health of middle-aged and older people.

**Additional Information.:**

Please access these Web sites via the online version of this summary at http://dx.doi.org/10.1371/journal.pmed.0050012.

- The MedlinePlus encyclopedia contains a page on healthy living (in English and Spanish)
- The MedlinePlus page on seniors' health contains links to many sites dealing with healthy lifestyles and longevity (in English and Spanish)
- The European Prospective Investigation into Cancer and Nutrition (EPIC) study is investigating the relationship between nutrition and lifestyle and the development of cancer and other chronic diseases; information about the EPIC-Norfolk study is also available
- The US Centers for Disease Control and Prevention provides information on healthy aging for older adults, including information on health-related behaviors (in English and Spanish)
- The UK charity Age Concerns provides a fact sheet about staying healthy in later life
- The London Health Observatory, which provides information for policy makers and practitioners about improving health and health care, has a section on how lifestyle and behavior affect health

## Introduction

A huge body of evidence indicates that lifestyles such as smoking, diet, and physical activity have a major influence on health [1–16]. However, achievable behavioural changes are often believed to have limited impact at an individual level. Nevertheless, a recent report from 2,339 men and women aged 70–90 y in 11 European countries indicated that adherence to a Mediterranean diet, nonsmoking, any alcohol use, and moderate physical activity were associated with more than 50% lower rate of all-cause and cause-specific mortality [6]. An advantage of an Europe-wide study is the great diversity in diet and other lifestyles [17,18], but one issue is whether such mortality differences can be observed in a single, relatively homogenous population within the usual range of lifestyle variations that may be more realistically achievable and directly relevant to immediate public health.

Additionally, assessment of diet and physical activity in most studies usually involves complex methodological analyses [6,16], and simpler indicators might be more feasible to use in estimating the potential combined impact of behavioural changes.

We have previously reported that high fruit and vegetable intake, as indicated by plasma vitamin C concentrations, predicts lower all-cause mortality in men and women [19]. We have also previously shown that low work and leisure-time physical activity predicts all-cause mortality and cardiovascular disease incidence [20]. Many health behaviours such as smoking habit, diet, and physical activity are highly correlated and, in aetiologically focused papers, treated as covariates. In the current analysis, we wished to explore the potential magnitude of their combined impact.

We examined the relationship between lifestyle using a simple health behaviour score based on smoking, physical activity, alcohol drinking, and fruit and vegetable intake, and total mortality by cause in men and women aged 45–79 y living in the general community.

## Methods

The participants were part of a prospective population study of 25,639 men and women aged 45–79 y, 99.5% white (as self-defined on questionnaire), resident in Norfolk, UK, first surveyed in 1993–1997. (Norfolk is a county in the UK encompassing a wide socioeconomic and urban-rural distribution.) They were recruited from age-sex registers of general practices as part of a ten-country collaborative study, the European Prospective Investigation into Cancer and Nutrition (EPIC). As virtually 100% of people in the UK are registered with general practitioners through the National Health Service, the age-sex registers form a population-based sampling frame. From the inception of the EPIC-Norfolk cohort, data collection was broadened to enable the examination of a wider range of determinants of chronic diseases. The Norfolk cohort was comparable to national population samples with respect to characteristics including anthropometry, blood pressure, and lipids, but with a lower prevalence of current smokers [21].

At the 1993–1997 baseline survey, participants completed a detailed health and lifestyle questionnaire. They were asked about medical history with the question “Has a doctor ever told you that you have any of the following?” followed by a list of conditions that included heart attack, stroke, and cancer. Smoking history was derived from yes/no responses to the questions “Have you ever smoked as much as one cigarette a day for as long as a year?” and “Do you smoke cigarettes now?” Alcohol consumption derived from the question “How many alcoholic drinks do you have each week?” with four separate categories of drinks. A unit of alcohol (approximately 8 g) was defined as a half pint of beer, cider, or lager; a glass of wine; a single unit of spirits (whisky, gin, brandy, or vodka); or a glass of sherry, port, vermouth, or liqueurs. Total alcohol consumption was estimated as the total units of drinks consumed in a week. For these analyses, a moderate drinker was defined as someone who drank one or more units a week (that is, not a nondrinker), but not more than 14 units a week.

Habitual physical activity was assessed using two questions referring to activity during the past year. The first question asked about usual physical activity at work, classified as four categories: sedentary, standing (e.g., hairdresser or guard), physical work (e.g., plumber or nurse), and heavy manual work (e.g., construction worker). The second question asked about the amount of time spent, in hours per week, in winter and summer in other physical activity. The average time spent daily in recreational activity was estimated as the total hours spent per week (average of winter and summer) in cycling and other physical activity such as swimming or jogging, divided by seven. A simple index allocated individuals to four ordered categories: inactive (sedentary job and no recreational activity); moderately inactive (sedentary job with <0.5 h recreational activity per day, or standing job with no recreational activity); moderately active (sedentary job with 0.5–1 h recreational activity per day, or standing job with <0.5 h recreational activity per day, or physical job with no recreational activity); and active (sedentary job with >1 h recreational activity per day, or standing job with >1 h recreational activity per day, or physical job with at least some recreational activity, or heavy manual job). This index was validated against heart rate monitoring with individual calibration in two independent studies [22,23]. We have also previously reported that this four-point index is inversely related to all-cause mortality and cardiovascular disease incidence in the EPIC-Norfolk population in men and women across a wide age and social class range [20]. For the purposes of the current study, we dichotomised the population into physically inactive (sedentary job and no recreational activity) and not physically inactive (any category with activity levels above the latter).

Social class was classified according to the Registrar General's occupation-based classification scheme into five main categories, with social class I representing professionals, social class II managerial and technical occupations, social class III subdivided into nonmanual and manual skilled workers, social class IV partly skilled workers, and social class V unskilled manual workers. We also recategorized social class into manual and nonmanual social classes. Social classes I, II, and III nonmanual were classified as nonmanual, whereas social classes III manual, IV, and V were classified as manual.[24].

Trained nurses carried out a health examination at a clinic. Height and weight were measured with subjects in light clothing without shoes. Body mass index was estimated as weight in kilograms divided by height in meters squared. Blood was taken by venepuncture into plain and citrate bottles. After overnight storage in a dark box in a refrigerator at 4–7 °C, they were spun at 2,100*g* for 15 min at 4 °C, and plasma and serum samples obtained. Six months after the start of the study, when funding became available, samples from participants were additionally taken for vitamin C assays. Plasma vitamin C was measured from blood drawn into citrate bottles. Plasma for vitamin C was stabilized in a standardized volume of metaphosphoric acid stored at −70 °C. Plasma vitamin C concentration was estimated using a fluorometric assay within 1 wk of sampling [25]. The coefficient of variation was 5.6% at the lower end of the range (mean, 33.2 μmol/l) and 4.6% at the upper end (mean, 102.3 μmol/l). We have previously reported that high plasma vitamin C level is inversely associated with mortality from all causes. Because humans do not manufacture vitamin C and have to rely on exogenous sources, plasma vitamin C is a good biomarker of plant food intake; previous studies have reported that a blood value of 50 mmol/l or more indicates an intake of at least five servings of fruit and vegetables daily [19;26].

We constructed a simple pragmatic health behaviour score. Participants scored one point for each of the following health behaviours: current nonsmoking, not physically inactive, moderate alcohol intake (1 to 14 units a week), and plasma vitamin C level >50 mmol/l, indicating fruit and vegetable intake of at least five servings a day. Participants could therefore have a total health behaviour score ranging from zero to four (Table 1). These particular health behaviours and their categorization were chosen based on extensive previous evidence on the relationship between these lifestyle factors and health endpoints.

**Table 1:**
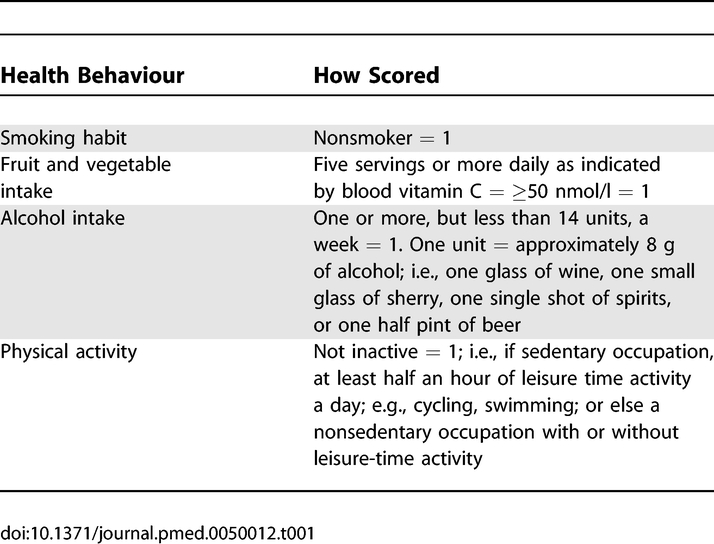
Health Behaviour Score: Score One Point for Each of the Health Behaviours Below for a Total Score of Zero to Four

All participants are followed up for health events. We report results for follow-up to July 2006, an average of 11 y. All participants are flagged for death certification at the Office of National Statistics, United Kingdom which is virtually complete. Death certificates for decedents are coded by trained nosologists according to the International Classification of Disease (ICD). Cardiovascular death was defined as those who had ICD 400–438 (ICD9) or ICD I10–I79 (ICD 10) as underlying cause of death and encompasses stroke and coronary heart disease as well as other vascular causes. Cancer death was defined as those who had ICD 140–208 (ICD9) or ICD C00–C97 (ICD 10) as underlying cause of death. Deaths not due to cardiovascular or cancer were classified as deaths from other causes. The study was approved by the Norwich District Health Authority Ethics Committee, and all participants gave signed informed consent.

The present analysis included all men and women aged 45–79 y who completed the health and lifestyle questionnaire and attended the health examination, who had complete data for physical activity, alcohol intake, and plasma vitamin C. Of the 22,301 with available data, 2,057 had a history of heart disease, stroke, or cancer at the baseline visit and were excluded from the main analyses, leaving 20,244 individuals.

We examined risk factor distributions in men and women. The Cox proportional hazards model was used to determine the relative risks of all-cause and cause-specific mortality by each of the individual health behaviours: current smoking, physical activity, moderate alcohol intake, and plasma vitamin C category after adjusting for age, sex, body mass index, and social class. We then examined mortality rates and relative risks of all-cause and cause-specific mortality by health score, adjusted for age, sex, body mass index, and social class. We estimated the difference in survival between those with health behaviour score of four compared to zero in age-equivalent terms by comparing the beta coefficient for mortality associated with each year of age with the beta coefficient difference in mortality for those with a score of four compared to zero [27]. We also examined relative risks in subgroups, stratified by sex, age group (<65 y and ≥65 y), body mass index category (<27 kg/m^2^ and ≥27/kg^2^), and manual and nonmanual social class, and also after excluding those who died within 2 y of follow-up. We additionally examined the relationship between health behaviour score and mortality in the 2,057 individuals with prevalent disease excluded from the main analyses.

## Results

Table 2 shows characteristics of the participants at baseline survey and mortality rates by cause after follow-up to 2006.

**Table 2:**
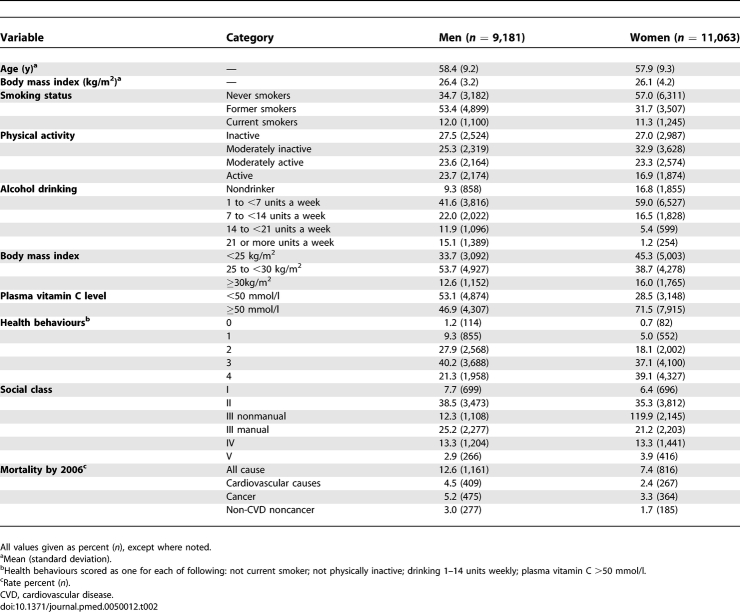
Distribution of Variables in 20,244 Men and Women Aged 45–79 y without Known Cardiovascular Disease or Cancer in EPIC-Norfolk at Baseline 1993–1997 and Mortality after Follow-Up to 2006 (Average 11 y)

Table 3 shows the relative risks for individual health behaviours by cause, adjusted for sex, body mass index, and social class. Each of the health behaviours: smoking, being physically inactive, not having a moderate alcohol intake, and a low fruit and vegetable intake as indicated by plasma vitamin C level <50 mmol/l. were associated with significantly higher risks of mortality from all causes. As might be expected, there were some differentials in the observed risk reductions observed for different health behaviours and cause-specific mortality in men and women; current smoking was the most consistent and strongest risk factor.

**Table 3:**
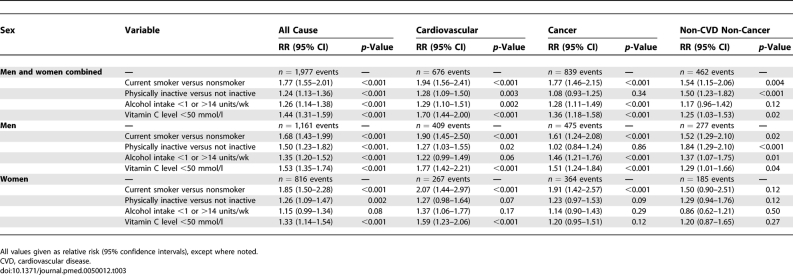
Independent Relative Risk (RR) of Mortality for Individual Health Behaviours by Cause, Adjusted for Age, Sex, Body Mass Index, and Social Class in 20,244 Men and Women Aged 45–79 y without Known Cardiovascular Disease or Cancer in EPIC-Norfolk 1993–2006, Cox Regression Model

Table 4 shows the relative risks for cause-specific mortality by number of health behaviours, adjusted for age, sex, body mass index, and social class. Risk of total mortality significantly increased with decreasing number of health behaviours, with a strong trend observed. Those who scored zero for the health behaviours had a relative risk of 4.04 (95% confidence interval [CI] 2.95–5.54) compared to those with a score of four. The greatest risk differences were observed for deaths attributed to cardiovascular diseases (relative risk [RR] 5.02; 95% CI 2.93–8.61) for score 0 versus score 4. Table 3 also shows that the trends were significant and consistent for all-cause mortality stratified by sex, age group <65 and ≥65 y, body mass index <27 and >27 kg/m^2^, manual and nonmanual social class, and after excluding deaths in the first 2 y. None of the interaction terms for health score with sex, age, body mass index, and social class were significant in multivariate analyses. In this cohort, vitamin supplement use was not associated with mortality, and results were similar after adjusting for vitamin supplement use or excluding vitamin users from the analyses (unpublished data and [19]). Table 5 shows the relative risks for cause-specific mortality by number of health behaviours in the 2,057 individuals with prevalent chronic disease not included in the main analyses. Results were very similar to those observed in individuals without known prevalent disease.

**Table 4:**
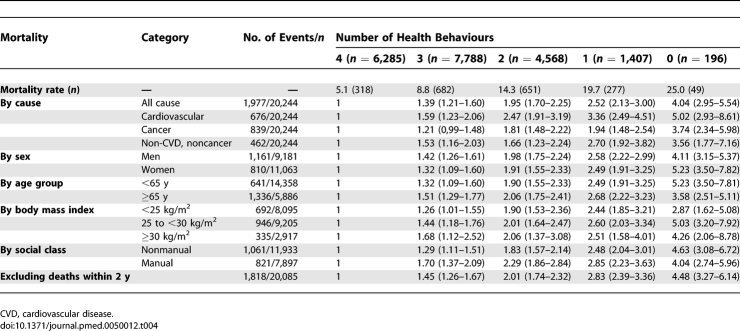
Mortality Rates and Relative Risk of All-Cause Mortality by Number of Health Behaviours, Adjusted by Age, Sex, and Body Mass Index, and Stratified by Cause, Sex, Age, Body Mass Index, and Social Class in 20,244 Men and Women Aged 45–79 y without Known Cardiovascular Disease or Cancer in EPIC-Norfolk 1993–2006, Cox Regression Model

**Table 5:**
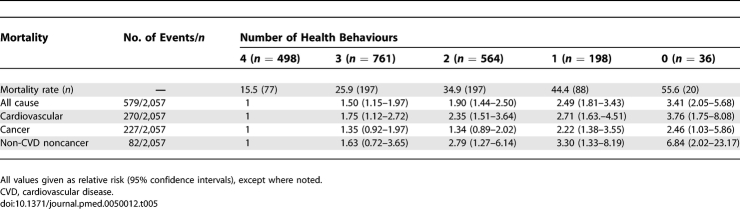
Mortality Rates and Relative Risk of All-Cause Mortality by Number of Health Behaviours, Adjusted by Age, Sex, and Body Mass Index, and Stratified by Cause, Sex, Age, Body Mass Index, and Social Class in 2,057 Men and Women Aged 45–79 y with Self-Reported Cardiovascular Disease or Cancer in EPIC-Norfolk 1993–2006, Cox Regression Model

**Figure 1:**
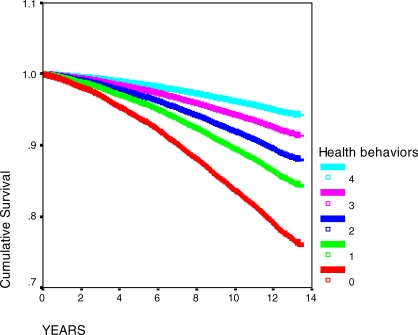
Survival Function According to Number of Health Behaviours in Men and Women Aged 45–79 Years without Known Cardiovascular Disease or Cancer, Adjusted for Age, Sex, Body Mass Index and Social Class, EPIC-Norfolk 1993–2006

Figure 1 shows survival curves over the average 11 y of follow-up, adjusted for age, sex, and body mass index by health score. As with the relative risks of mortality, the adjusted cumulative survival was about 75% for those scoring zero and 95% for those scoring four, respectively, for health behaviours. From the Cox model, the beta coefficient for mortality associated with each year increase in chronological age was 0.10 (± standard error 0.004). The difference in beta coefficients between a health score of zero versus four was 1.43, that is, equivalent to approximately 14 y in chronological age for mortality risk.

## Discussion

In these middle-aged and older men and women, four health behaviours—not smoking, not being physically inactive, having a moderate alcohol intake (1–14 units a week), and having a high fruit and vegetable intake (as indicated by plasma vitamin C level >50 mmol/l)—were combined into a simple pragmatic four-item health behaviour score that was inversely related with mortality over an average 11 y of follow-up. There was a strong trend of decreasing mortality risk with increasing number of positive health behaviours, with those who scored four having approximately one quarter the mortality risk of those who scored zero, equivalent to about 14 y difference in chronological age. Although the trends were strongest for deaths from cardiovascular causes, they were also apparent for deaths from cancer and from other causes. They were also consistent after stratifying by sex, age group, body mass index, and social class, and after exclusion of deaths in the first 2 y. In the individuals with prevalent disease who were not included in the main analyses, we also found similar trends in mortality with the health behaviour score.

The evidence that behavioural factors such as diet, smoking, and physical activity influence health is overwhelming. However, these health behaviours are usually highly correlated, and only recently have these factors been examined in combination. Chiuve et al. reported that in men in the US Health Professionals Study, men with five low-risk health behaviours, that is nonsmokers, with a body mass index <25 kg/m^2^, moderate to vigorous activity, moderate alcohol consumption, and the top 40%of a healthy diet score had a 0.13 risk of coronary heart disease compared to men who did not adhere to any of these behaviours [2]. Our estimates with comparable measures for smoking, alcohol, and physical activity, but with a simpler diet measure, are comparable for deaths from cardiovascular causes. Whether combined lifestyle factors are also related to other diseases or all-cause mortality has been less well documented till recently. Knoops et al. reported that in 2,339 men and women aged 70–90 y in 11 European countries, the combination of four factors—adherence to a Mediterranean diet, moderate alcohol use, being physically active, and nonsmoking—was associated with a mortality rate one third of those who did not have these behaviours [6]. As Rimm and Stampfer have pointed out, these results are consistent with studies suggesting similar substantial reductions in risk of chronic diseases such as coronary heart disease, diabetes, and cancer associated with lifestyle behaviours [28]. However, as Rimm and Stampfer and others have also highlighted, the Knoops study was conducted on a highly selected older group of individuals in 11 different European countries with very different mortality rates, and the generalisability of these results to younger populations is uncertain [17,18]. It also did not have the power to examine the consistency of findings within subgroups, for example, stratifying by sex or obesity. Findings from the current study support those from previous reports in more diverse populations: even within the range of usual lifestyle in a free-living, relatively homogenous population living in one region of UK, there were substantial differences in mortality associated with the four health behaviours combined, and these differences were consistent in several population subgroups stratified by sex, age, social class, and obesity.

Additionally, many studies that have reported on diet and physical activity have used detailed complex instruments for assessment of these lifestyles, to obtain for example, a Mediterranean diet score or a physical activity score [6,16]. These instruments are useful for research purposes, but a simpler, more pragmatic health behaviour score may be more easily used for clinical or public health practice. We also wished to examine the relationship with mortality and consistency over a wide range of different groups in the population stratified by sex, age, body mass index, and social class. The score, though simple, was based on instruments that have been extensively previously validated. We used plasma vitamin C as that has been previously shown to be a good biomarker of fruit and vegetable intake, and the association between blood biomarker and dietary intake well quantified. In this cohort, vitamin supplement use was not associated with mortality, and results were similar after excluding those using vitamin supplements. Since many dietary practices are highly correlated, it may also be a surrogate marker for particular dietary patterns such as high fibre intake, or low fat intake that may have additional health effects. Although the recent Women's Health Initiative reported that women in the dietary intervention arm did not have significantly lower cardiovascular endpoints and nonsignificant differences for breast cancer, explanations for the lack of effect have been extensively discussed elsewhere, including smaller dietary differences between control and intervention arms than originally planned [29–31]. Nevertheless, there is a large body of experimental and epidemiologic evidence indicating a high intake of fruit and vegetables is beneficially associated with health [5,7,11,32] Similarly, the simple physical activity score used here has been extensively validated as a measure of total energy expenditure and also predicts total mortality and cardiovascular disease incidence.

There is also a large body of evidence relating alcohol intake to mortality. There is some debate about the nature of the relationship, with the general consensus of a U-shaped relationship; with nondrinkers and heavy drinkers being at increased risk. Internationally, upper-limit recommendations for alcohol intake range from maximum of five drinks daily for men and three drinks daily for women in France to two drinks daily for men and one for women in the United States. In the UK, the recommendations are up to 21 drinks weekly for men and 14 drinks weekly for women [33]. We used a generally accepted definition of moderate drinking as at least one drink a week, but not more that 14 drinks a week, with the upper end well within the generally recommended upper range.

It is possible that people who are already ill may be more likely to be physically inactive and change their diet as a result of prevalent disease. However, individuals with known serious chronic disease, namely cancer, heart disease, and stroke, were excluded from the main analyses. Nevertheless, even in those individuals with known diseases, subsequent survival was also strongly related to health behaviour score. Additionally, the relationships were consistent after excluding all those who died within 2 y of the baseline and after stratification for major potential confounders such as age, obesity, and social class. Though we cannot exclude residual confounding, our results are consistent with the existing evidence indicating these behavioural factors are beneficial for health. Any potential unknown confounders would have to explain plausibly the substantial differences in mortality risk. In these particular analyses, we did not examine how far, if at all, the behavioural associations were mediated through classical cardiovascular risk factors, though previous analyses have suggested these are independent. Nevertheless, the magnitude of the behavioural associations are substantially greater than those reported for many individual physiological risk factors such as blood pressure, lipids, or C-reactive protein, such that they are likely to act synergistically on several different biological pathways.

This study has several limitations. There are potential large measurement errors in the assessment of exposures. We used only a measure at one point in time to characterize individuals and did not take into account likely changes in lifestyles over the follow-up period. Nevertheless, random measurement error is likely to attenuate any associations observed, so the estimated differences in risk are likely to be larger than those observed. Secondly, though clearly different health behaviours differ somewhat in their association with different endpoints, we did not weight them because the aim of the current approach was to examine the use of a simple score that could be conceptually easy to understand and use in clinical practice, rather than complicated algorithms. Nevertheless, the simple score was strongly related with mortality; imprecision is likely again only to attenuate any relationships. Thirdly, the proportions of the population with some or all positive health behaviours were relatively high since the definitions for health behaviours were not necessarily optimal, for example, for physical activity [20], and dichotomizing behaviours between inactive and not inactive may have obscured the gradient in mortality between those who were moderately inactive and those who were active. Nevertheless, this demonstrates that the behaviours associated with substantial differences in mortality risk are entirely feasible and achievable by most of the population.

### Implications

Our data examined only mortality. With ageing populations, a major challenge is not just premature mortality, but functional health, which relates to quality of life. Nevertheless, we have also previously reported that these lifestyle factors are also associated with similar substantial differences, with subjective functional health of comparable magnitude [34;35], and subjective functional health is also predictive of mortality [36]. The four health behaviours were within the usual range found in a free-living population. Though relatively modest and achievable, their combined impact was associated with an estimated 4-fold difference in mortality risk, equivalent to 14 y in chronological age. Notably, the differences in survival were also observed in people with existing chronic disease. These results may provide further support for the idea that even small differences in lifestyle may make a big difference to health in the population and encourage behaviour change.

## Abbreviations

CI: confidence interval
ICD: International Classification of Disease
RR: relative risk
